# Toll-Like Receptors and Skin Cancer

**DOI:** 10.3389/fimmu.2014.00135

**Published:** 2014-03-31

**Authors:** Erin M. Burns, Nabiha Yusuf

**Affiliations:** ^1^Department of Dermatology, University of Alabama at Birmingham, Birmingham, AL, USA

**Keywords:** Toll-like receptor, non-melanoma skin cancer, BCC, SCC, melanoma, innate immunity

The skin, the largest organ in the body, provides the first line of defense against the environment both as a physical barrier and as a key immunological component. Toll-like receptors (TLRs) serve as signaling molecules that recognize pathogen-associated molecular patterns (PAMPs) as well as damage-associated molecular patterns (DAMPs), and are expressed by various skin cells including keratinocytes and melanocytes, which are the main cell types involved in both non-melanoma and melanoma skin cancers. TLRs induce inflammatory responses meant for clearing pathogens, but can ultimately contribute to skin carcinogenesis. In contrast, TLR agonists, specifically targeting TLR7, 8, and 9, have been successfully used as therapeutics for melanoma and basal cell carcinoma (BCC), functioning by recruiting dendritic cells and inducing T-cell responses. Here, we discuss the role TLRs play in skin carcinogenesis as well as the use of TLRs as targets for skin cancer treatment options.

## Skin and TLRs

Non-melanoma skin cancer (NMSC) includes BCC and squamous cell carcinoma (SCC). With over 3.5 million new diagnoses annually, NMSC is the most common cancer in the United States (1). Risk factors for developing NMSC include ultraviolet (UV) light exposure, skin color, sunburns, age, and immunosuppressive status (2). NMSCs account for over 3,000 deaths each year (3) and also contribute to over $1.4 billion annually for the treatment and management of these skin tumors (4). Melanoma contributes to approximately 5% of all skin cancer diagnoses, with 76,000 new cases diagnosed in 2012 (5). Importantly, melanoma leads to over 9,000 deaths annually, which accounts for the majority of skin cancer deaths. Risk factors for melanoma include UV exposure, sunburn, nevi, immunosuppressive status, and family history.

The most common treatments for SCC include excision, Mohs micrographic surgery, and cryosurgery, which, when the lesion is detected early and promptly removed, are effective and cause minimal damage (2). If left untreated, the tumors are able to grow exponentially or metastasize, leading to more invasive procedures. For melanoma, surgical excision is the most common treatment, with recent preferences for Mohs surgery (5). However, in the case of recurring lesions or lesion patches, surgery may not be an option due to extensively damaged skin or lack of tissue for removing clear margins, resulting in the need for alternative treatment options.

The skin is the largest organ in the body and contains three major cell types, which include melanocytes, Langerhans cells, and keratinocytes. Keratinocytes are the major cell type of the epidermis and provide defense against the environment both as a physical barrier and a key component of the innate immune response (6, 7). Epidermal keratinocytes, as the outmost environmental barrier, are responsible for the production of antimicrobial peptides (8), which are up-regulated by various stimuli through both the mitogen-activated protein (MAP) kinase and nuclear factor (NF) kappaB pathways (9). TLRs are expressed by various skin cells including keratinocytes and melanocytes (10), which are the main cell types involved in both non-melanoma and melanoma skin cancers. Human keratinocytes have been shown to express TLRs 1–6 and 9 (10–14). Recently, it has been reported that TLR2–5, 7, 9, and 10 are constitutively expressed in human melanocytes (15).

Toll-like receptors serve as signaling molecules that recognize PAMPs, or pathogen-associated molecular patterns, as well as DAMPs and thus, activate the innate immune response through the transcription factor NF-kB (16). The 10 human TLR family members are characterized by the leucine-rich repeat domain content in both their extracellular region and the intracellular Toll-IL-1 receptor (TIR) domain (17), which can therefore interact with adaptor molecules that contain appropriate adaptor molecules (18).

Toll-like receptors have been demonstrated to be important for both innate immune response specificity (19, 20) as well as for adaptive immune responses such as dendritic cell maturation and costimulatory molecule expression and the promotion of Th-1 cell-mediated responses through increased production of IL-12 by activated TLRs on dendritic cells (21, 22). It also has been reported that innate inflammatory responses localized to the epidermis may be affected by TLR expression in human melanocytes (23). TLRs are activated in melanocytes, as a consequence of the inflammatory response to tissue injury, sunburn or skin infection, and constitute a natural defense to recruit innate immune cells.

## TLR Stimulation and Skin Carcinogenesis

Besides their function of recognizing exogenous PAMPs, TLRs also recognize endogenous ligands, which are often referred to as alarmins and function to recognize cell or tissue damage and alert the innate and adaptive immune systems (24, 25). Expression association studies have revealed potential functions of TLR endogenous ligands in tumorigenesis. For example, high-mobility group box-1 protein (HMGB1) can function as a DAMP and is released in response to tissue or cellular damage. It is over-expressed in several human neoplasms including lung, pancreatic, breast, liver, and colorectal cancers, and, importantly, melanoma (26). HMGB1 is either passively released by injured or necrotic cells (27) or actively secreted by monocyte/macrophages, neutrophils, and dendritic cells [reviewed in Ref. (28)].

With the exception of TLR3 that signals through Toll/IL-1R domain containing adaptor inducing IFN (TRIF), TLRs signal through myeloid differentiation factor 88 (MyD88). TLR signaling has been reviewed extensively elsewhere (29). MyD88 is an adaptor protein that is ultimately responsible for initiating NF-kB activation (30), and therefore the amplification of inflammation and the promotion of tumor development (31). Importantly, chronic inflammation has been linked to tumor development in animal models of both spontaneous and chemically induced carcinogenesis (32, 33).

Tumor cells expressing TLRs may be able to evade immune surveillance processes, thus promoting tumor development. The activation of TLR4 and subsequent signaling molecules have been shown to upregulate immunosuppressive cytokines such as IL-10 as well as pro-inflammatory cytokines and chemokines including IL-6, IL-18, and TNF-α, which have been shown to contribute to tumor development, growth, and even metastasis (34). In human melanoma A375 cells, the inhibition of TLR4/MyD88 signaling effectively decreased both VEGF and IL-8 levels with paclitaxel and icariside II combination treatment (35). TLR2-4 are expressed and up-regulated in several human metastatic melanoma cell lines (36), with recent data indicating that melanoma cells also express TLR7, 8, and 9 (37), which are abnormally up-regulated in cells from melanoma biopsies (38). The over-expression of TLR4 within melanoma tumors triggers an inflammatory response leading to tumor development (39). TLR9 activation has also been shown to enhance invasion as well as promote proliferation in several cancer cell lines via NF-kB and Cox-2 activation (40), as well as the secretion of IL-8 and IL-1α (41), and TGF-β (42). Recent studies in head and neck cancer have revealed that TLR3 expression and signaling affects the migration and metastatic potential of tumors as evidenced in oral SCC by inducing CCL5 and IL-6 secretion (43).

Importantly, TLR inhibition can exert anti-cancer effects. TLR4 pathway inhibition reversed tumor-mediated suppression of both natural killer cell activity as well as T-cell proliferation *in vitro* and *in vivo*, resulting in increased tumor latency and survival of tumor-bearing mice (44). TLR2 plays an important role in the induction of tumor regression, which has been demonstrated in a mouse model of glioblastoma multiforme where blocking HMGB1-mediated TLR2 signaling via tumor-infiltrating myeloid DCs resulted in a loss of therapeutic efficacy (45).

TLR3 activation on immune cells results in anti-cancer activities, where T cell-mediated responses are promoted (46). Specifically, upon stimulation with TLR3 agonist poly(I:C), CD8 T cell responses are enhanced, leading to the production of IFNγ and TNF-α and ultimately, the generation of memory CD8 T cells.

## TLR-Targeted Therapy

Although TLR expression on tumor cells may allow tumors to evade surveillance, TLRs are also considered to be targets for anti-cancer interventions that result in the recognition and ultimate destruction of tumor cells using a tolerant immune system. This idea is further illustrated by the fact that recent studies have demonstrated a dual nature of immune responses in the context of cancer therapies, highlighting the importance of considering conditions, TLR targets, and combinations of immune interventions and TLR ligands (47).

There are studies and case reports that show that 5% imiquimod cream treatment is an effective therapeutic option for actinic keratosis (AK), BCC, Bowen’s disease, and lentigo maligna (48–53). The mechanism of action of imiquimod is through the activation of TLR7 (54), and imiquimod has been approved to treat both premalignant actinic keratoses, and malignant superficial BCC (55). The mechanism may also involve Th1-response promotion, the recruitment of macrophages, anti-tumor cytotoxic CD8 T cells, and NK cells to the lesion, as well as induce apoptosis of tumor cells (55, 56). Imiquimod has also been shown to induce IFN-α and IL-12 production, resulting in a heightened immune response (49, 57, 58). The suggested mechanism for exertion of anti-tumor effects on UVB-induced SCC by imiquimod is through the activation of Th17/Th1 cells as well as cytotoxic T lymphocytes (59). Five percent topical imiquimod has been effective in several clinical trials (49, 53, 57, 60). The related drug, resiquimod, has been demonstrated as a safe and effective topical intervention for AK and is a potential treatment option for patients who have large patches of AK (61).

Several cancer types including melanoma have been successfully treated with Taxol, CpG, or otherTLR ligands (62, 63). PF3512676, a synthetic CpG ODN, uses a TLR9-targeted approach to effectively treat BCC (64). TLR 7 and 8 agonists activate a pro-inflammatory response for SCC treatment (65). Additionally, IL-1, 6, 8, and 12 modulation along with a promotion of a Th1-response have been shown to exert anti-tumor and antiviral behavior (65).

Previous studies have demonstrated TLR3 agonists to be promising adjuvants for cancer vaccines, especially in regards to their immunostimulatory properties (46). A recent study has demonstrated that human melanoma cells express TLR3, which in combination with TLR3 agonists, results in tumor cell death via caspase activation when cells are pretreated with cycloheximide or IFN-α (38), suggesting that TLR3 agonists may be multifunctional adjuvants offering more clinical treatment options. Therefore, TLRs and their signaling pathways may be potential therapeutic targets to control tumor progression, especially in diseases such as cutaneous malignant melanoma, which is an aggressive tumor that is not effectively managed with current treatments (66).

It is important to note that, especially in the case of TLR7 agonists such as imiquimod and resiquimod, though quite effective when applied topically to AKs and BCCs, systemic therapeutic interventions have not been as successful. This TLR tolerance has previously been demonstrated with TLR4 agonists, which resulted in decreased NF-kB activation (67). The suggested mechanism for TLR7 tolerance is the diminished capacity for IL-12 secretion as well as IFN-α secretion by plasmacytoid DCs (68). Recent studies have found that local and systemic TLR-targeted therapies have different modes of action and require further investigation, especially into the timing and dosage of treatments to reach maximum efficacy without inducing TLR tolerance (69).

## Conclusion

In summary, TLRs are an important immunological component expressed by keratinocytes and melanocytes, which are the main cell types involved in both non-melanoma and melanoma skin cancers. TLRs induce inflammatory responses meant for clearing pathogens, but their activation can also potentiate chronic inflammation, which can ultimately contribute to skin carcinogenesis. In contrast, TLR agonists, specifically targeting TLR7, 8, and 9, have been successfully used as therapeutics for melanoma and BCC, functioning by recruiting dendritic cells and inducing T-cell responses. It is important to consider local versus systemic applications of TLR therapies and the balance between efficacy and inducing TLR tolerance. TLR3 agonists have been shown to be well-tolerated and effective in both directly killing cancer cells and directing immune responses in melanoma. TLR-targeted therapies may be potential treatment options for large or reoccurring skin tumors that may be difficult to treat with surgery or for other skin tumors that are not responsive to current therapies.
